# Metagenomic Insights into the Anti-Obesity Effect of a Polysaccharide from *Saccharina japonica*

**DOI:** 10.3390/foods12030665

**Published:** 2023-02-03

**Authors:** Ying Song, Dongze Lu, Honggang Wang, Zhenyi Zhou, Xian Luo, Manjing Ma, Songze Ke, Hong Wang, Yanlei Yu, Bin Wei

**Affiliations:** Key Laboratory of Marine Fishery Resources Exploitment & Utilization of Zhejiang Province, College of Pharmaceutical Science & Collaborative Innovation Center of Yangtze River Delta Region Green Pharmaceuticals, Zhejiang University of Technology, Hangzhou 310014, China

**Keywords:** *Saccharina japonica*, polysaccharides, gut microbiota, obesity, 16S rRNA gene sequencing, metagenome-assembled genomes

## Abstract

*Saccharina japonica* polysaccharides exhibit great potential to be developed as anti-obesity and prebiotic health products, but the underlying mechanism has not been adequately addressed. In this study, we investigated the potential mechanism of a *S. japonica* polysaccharide fraction (SjC) in preventing high-fat-diet (HFD)-induced obesity in mice using 16S rRNA gene and shotgun metagenomic sequencing analysis. SjC was characterized as a 756 kDa sulfated polysaccharide and 16 weeks of SjC supplementation significantly alleviated HFD-induced obesity, insulin resistance, and glucose metabolism disorders. The 16S rRNA and metagenomic sequencing analysis demonstrated that SjC supplementation prevented gut microbiota dysbiosis mainly by regulating the relative abundance of *Desulfovibrio* and *Akkermansia*. Metagenomic functional profiling demonstrated that SjC treatment predominantly suppressed the amino acid metabolism of gut microbiota. Linking of 16S rRNA genes with metagenome-assembled genomes indicated that SjC enriched at least 22 gut bacterial species with fucoidan-degrading potential including *Desulfovibrio* and *Akkermansia,* which showed significant correlations with bodyweight. In conclusion, our results suggest that SjC exhibits a promising potential as an anti-obesity health product and the interaction between SjC and fucoidan-degrading bacteria may be associated with its anti-obesity effect.

## 1. Introduction

Obesity refers to the excessive accumulation or abnormal distribution of body fat. It is a chronic metabolic disease caused by the interaction of multiple factors including genetics and environment [1]. With the occurrence and aggravation of obesity, it will lead to a variety of metabolic-related diseases, such as insulin resistance, hypertension, diabetes, and heart disease [2]. The current global epidemic of obesity has become an important health problem faced by countries all over the world. Finding safe and effective ways to prevent obesity has become a research hotspot. Studies have shown that *Sargassum fusiforme* fucoidan (SFF) can improve high-fat-diet (HFD)-induced insulin resistance by repairing damaged Akt phosphorylation, reducing the level of MDA and 4-HNE-modified protein, and activating the Nrf2 signal pathway. At the same time, SFF supplementation reshapes gut microbiota composition and reduces intestinal inflammation [3]. Studies have shown that the gut microbiota can affect obesity by regulating energy intake, inhibiting energy metabolism, and inducing chronic inflammation [4,5,6].

*Saccharina japonica*, the most widely cultivated and consumed commercial edible seaweed in the world, has attracted remarkable attention in recent years due to its important biological activities and highly diverse structures [7]. More and more studies have shown that polysaccharides prepared from *S. japonica* have anti-obesity, hypoglycemic, and antibacterial effects [8,9,10]. A low-molecular-weight polysaccharide isolated from *S. japonica* by enzymatic preparation significantly alleviated HFD-induced obesity through downregulating sterol regulatory element-binding protein and fatty acid synthase and suppressing lipid synthesis [11]. Sodium alginate extracted from brown seaweeds could effectively suppress obesity by regulating the expression of colonic lipid and carbohydrate metabolism [12]. However, the mechanism of *S. japonica* polysaccharides in preventing high-fat-diet (HFD)-induced obesity is still largely unclear.

Previous in vitro studies have shown that the gut microbiota can degrade *S. japonica* polysaccharides into oligosaccharides, which is more conducive to the proliferation of probiotics and the production of organic acids [13,14]. In vivo studies suggest that polysaccharides or some dietary fibers can be decomposed and utilized by the gut microbiota in the large intestine to produce the acidic metabolites, SCFAs, thereby reducing the pH of the intestinal environment and promoting the proliferation of probiotics [15,16,17]. Our recent study indicated that a 5.1 kDa fucan prepared from *S. japonica* could suppress high-fat-diet-induced obesity and enrich fucoidan-degrading gut bacteria [18]. Fucoidan can be used by the gut microbiota in human feces and may have the potential to improve intestinal health [19]. Studies have shown that fucoidan can improve oxidative stress and the inflammatory response [20]. However, a detailed genomic and functional examination of these bacteria is needed to clarify the exact mechanism of *S. japonica* polysaccharides in preventing obesity.

Here, a polysaccharide fraction (SjC) was prepared from *S. japonica* and its chemical structure was characterized using various spectroscopic techniques. Then, the effects of SjC in preventing high-fat-diet-induced obesity in mice and gut microbiota dysbiosis were investigated, and the potential mechanism of SjC in regulating bodyweight and the gut microbiota structure was explored through metagenomic sequencing and bioinformatic analysis. This study will provide novel insights for the mechanistic studies of seaweed polysaccharide in preventing obesity and contribute to the development of seaweed polysaccharides as dietary prebiotics.

## 2. Materials and Methods

### 2.1. Preparation of SjC from S. japonica

The brown algae, *S. japonica,* were collected in Rongcheng, China in November 2020. SjC was prepared according to a previous study [21]. Briefly, dry *S. japonica* was cut into pieces and extracted with 1% calcium chloride (Sinopharm Chemical Reagent Co., Ltd., Shanghai, China, AR) aqueous solution at 100 °C for 3 h and the crude extracts were filtered and concentrated. Then, the remaining supernatants were ultrafiltrated (2.5 kDa) to remove inorganic salts, monosodium glutamate sugars, and small molecular weight polysaccharides, and finally to obtain SjC.

### 2.2. Structural Analysis of SjC

According to our previous study, the monosaccharide composition of SjC was determined by pre-column derivatization with PMP (Sinopharm Chemical Reagent Co., Ltd., Shanghai, China, AR) and the molecular weight was determined by a gel permeation chromatography–high performance liquid chromatography (GPC–HPLC) system (Waters e2695, Waters, MA, USA). The sulfate content was detected according to the barium chloride–gelatin method [22]. NMR spectra were recorded on a Bruker AVANCE III 600 MHz (Billerica, MA, USA) at 25 °C with sample preparation similar to the previous study [18]. An amount of 1 mg of polysaccharide sample was added to 99 mg of dry KBr powder (Sinopharm Chemical Reagent Co., Ltd., Shanghai, China, AR), mixed uniformly, pressed into tablets, and scanned at a wavelength of 400–4000 cm^−1^ using a FT-IR spectrophotometer (Infrared Spectrometer TRENSOR 27, Bruker Daltonics, Ettlingen, German).

### 2.3. Animal Experiment

SPF-grade male C57BL/6J mice (20–25 g) purchased from SPF (Beijing) Biotechnology Co., Ltd. (Beijing, China) were housed in the temperature control room (24 ± 1 °C), light/dark cycle for 12 h. All mice were fed standard laboratory chow for one week for acclimatization and then randomly divided into three groups (Control, Model, and SjC groups, *n* = 5–6 per group). After acclimation, mice in the Model and SjC groups were fed with a HFD diet (TP23301, 60% kcal fat, Trophic Animal Feed High-Tech Co., Ltd., Nantong, China) and mice in the Control group were fed with a low fat diet (TP23303, 10% kcal fat, Trophic Animal Feed High-Tech Co., Ltd., Nantong, China). The mice in the SjC group had free access to drinking water supplemented with SjC solution (1 mg/mL) for 16 weeks and the other two groups were freely given common drinking water. During the experiment, the food consumption of mice was measured every two days and the weight of mice was measured every week. After the experiment, the mice fasted for 12 h and were euthanized with carbon dioxide. The blood collected by cardiac puncture was centrifuged at 3000 rpm/min at 4 °C for 10 min to obtain the serum. Liver and epididymal fat were collected and weighed. Cecal contents were collected and stored at −80 °C. All animal experiments comply with the regulations of the Ethics Committee for Animal Research of Zhejiang University of Technology (201908547).

### 2.4. Biochemical Measurements

OGTT and ITT were measured at 14 and 15 weeks, respectively, according to our previous reports [23]. Total cholesterol (TC) and triglycerides (TG) were determined using commercially available biochemical kits purchased from Nanjing Jiancheng Bioengineering Institute (Jiangsu, China).

### 2.5. Gut Microbiota Analysis by 16S rRNA Gene and Shotgun Metagenomic Sequencing

According to the manufacturer’s instructions, the genomic DNA in the caecum contents of mice was extracted using the TIANAmp Stool DNA Kit (TIANGEN, DP328). The V3-V4 region of the 16S rRNA gene was amplified by the universal primers, 338F (5-CTCC-TACGGAGGCAGCA-3) and 806R (5-GGACTACHVGGGTWTCTAAT-3), and subjected to the Illumina Miseq PE250 platform for sequencing. The bioinformatics analysis was performed using QIIME2 pipelines [24]. For the shotgun metagenomic sequencing, DNA libraries were prepared using the Enzymic Universal DNAseq Library Prep Kit (KAITAI-BIO, AT4107) according to the instructions for use with an insert size of 350 bp. The DNA library was sequenced (150 bp, paired-end reads) on the Illumina NovaSeq 6000 platform. Quality control was performed using Fastqc 0.11.08 and host sequences were eliminated using BW A 0.7.17 [25]. The “-- metabat2 --maxbin2 --complex” parameter was used to assemble the quality control reading into the bins, then the bins were refined and reassembled through the “- c 50-x 10” parameter of metaWRAP 1.3.2, and finally, the metagenomic-assembled genomes (MAGs) were acquired. Homologous nucleotide similarity was calculated by the Orthologous Average Nucleotide Identity Tool 0.93.10 [26]. Finally, the MAGs were functionally annotated using humann 3 and annotated by EnrichM v0.6.4 (https://github.com/geronimp/enrichM, accessed on 27 August 2022), and their metabolic pathways were compared using the KEGG Orthology database.

### 2.6. Linking 16S rRNA Gene Sequences with Metagenome-Assembled Genomes

To explore if the function of gut bacteria was significantly affected by SjC treatment, we attempted to link the 16S rRNA gene sequences with the MAGs using BLAST analysis [27]. Briefly, all unique 16S rRNA gene sequences were blast against the genes annotated from the MAGs. Only when the length of the alignment is greater than 300 bp and the identity is greater than 99%, the 16S rRNA gene sequence and the MAG are considered to come from the same strain.

### 2.7. Prediction of Bacteria with Fucoidan-Degrading Potential

Previous studies demonstrated that at least twelve bacterial strains, including eight *Lentimonas* spp., *Mariniflexile fucanivorans* DSM 18792, and *Kiritimatiellales* sp. F21 [28], could degrade seaweed polysaccharides and our recent study indicated that the relative abundance of the main gene categories of these fucoidan-degrading bacteria was quite similar; therefore, the fucoidan degradation potential of bacteria was predicted by calculating the Jensen–Shannon divergence of the gene class distribution. The fucoidan degradation potential of MAGs was predicted by the Jensen–Shannon divergence.

### 2.8. Statistical Analysis

All data are expressed by mean ± SEM. Student’s *t*-test in GraphPad Prism 8.0 software was used to analyze the significance of differences between the two groups. One-way ANOVA and Tukey multiple comparison analysis were used to analyze the significance between multiple groups.

## 3. Results

### 3.1. Structure Analysis of SjC

SjC was prepared from *S. japonica* with calcium chloride and characterized as a 756 kDa sulfated polysaccharide. The main monosaccharide components of SjC were fucose and mannose (molecular ratio of 4.6:1), and SjC also contained a small amount of rhamnose and galactose (Figure S1). The chemical composition analysis showed that SjC contained 25.7% sulfate. The FT-IR results show that the absorption peak at 3437 cm^−1^ is the contraction vibration peak of OH and the weak absorption peak at 2938 cm^−1^ is the stretching vibration of C-H, including CH, CH_2_, and CH_3_ (Figure S2). The absorption peak is the stretching vibration peak of C=O and the absorption peak at 1266 cm^−1^ is generated by the stretching vibration of S=O, which is caused by sulfate radicals. The vibration at 1046 cm^−1^ is the characteristic signal of the glycosidic bond [29]. The ^1^H-NMR spectrogram was crowded in a cramped region ranging from 3 to 5 ppm, which confirmed the presence of polysaccharides [30,31] (Figure S3). Signals in the 5–6 ppm range suggest the presence of α-anomeric protons. A signal of 4.8 ppm is produced by D_2_O and 0.8–2.0 ppm is shown as the chemical shift value of CH_3_ (Figure S3).

### 3.2. SjC Supplementation Prevented HFD-Induced Obesity and Metabolic Disorders

**Figure 1:**
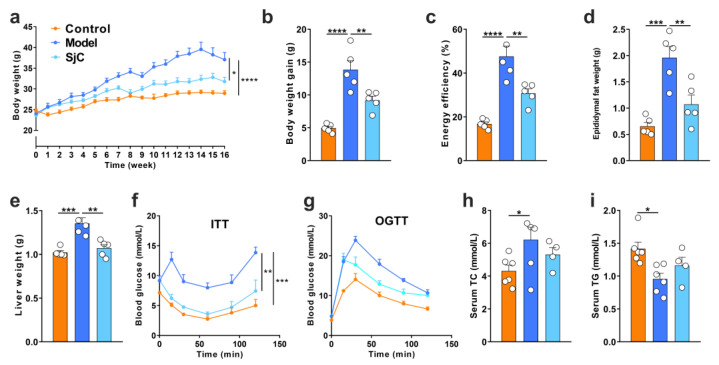
SjC supplementation prevented HFD-induced obesity and metabolic disorders. (**a**) Bodyweight. (**b**) Bodyweight gain. (**c**) Energy efficiency. (**d**) Epididymal fat weight. (**e**) Liver weight. (**f**) Blood glucose curves after intraperitoneal injection of insulin. (**g**) Blood glucose curves after an oral load of glucose. (**h**) Serum total cholesterol. (**i**) Serum triglycerides. All data are expressed by mean ± SEM (*n* = 3–6). One-way ANOVA and Tukey multiple comparison analysis were used to analyze the significance between multiple groups, * *p* < 0.05; ** *p* < 0.01; *** *p* < 0.001; **** *p* < 0.0001.

The effects of 16 weeks’ treatment with polysaccharide SjC on HFD-induced obesity and metabolic disorder were determined in C57BL/6J mice. According to Figure 1a,b, 16 weeks of HFD feeding resulted in severe bodyweight gain in mice compared with the normal group, but the SjC supplementation intervention effectively prevented HFD-induced weight gain. SjC can also effectively reduce the energy efficiency of mice (Figure 1c). Simultaneously, HFD feeding induced increases in epididymal fat (Figure 1d) and liver weight (Figure 1e) in mice, which were also alleviated by the SjC intervention. Furthermore, HFD feeding significantly aggravated glucose and insulin resistance in mice compared with the control group, and SjC supplementation significantly attenuated impaired insulin resistance (Figure 1f) and slightly improved abnormal glucose tolerance (Figure 1g). In addition, SjC slightly attenuated HFD-induced alterations of serum total cholesterol and triglyceride levels (Figure 1h,i), but the difference was not statistically significant.

### 3.3. SjC Supplementation Alleviated HFD-Induced Gut Microbiota Dysbiosis at Species Level

**Figure 2:**
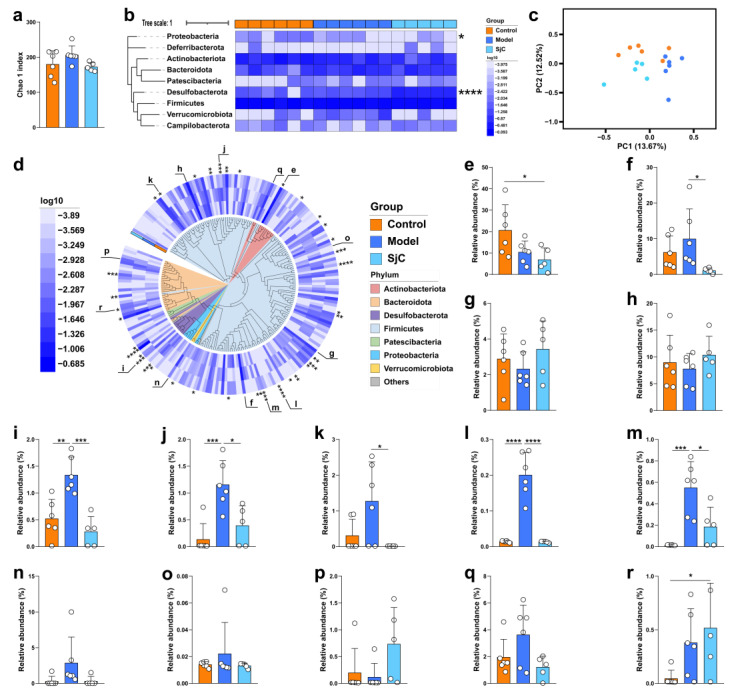
16S rRNA gene sequencing analysis revealed the effects of SjC administration on gut microbiota composition in HFD-fed mice. (**a**) Chao 1 index. (**b**) Gut microbiota composition at the phylum level. (**c**) Principal component analysis (PCA) of gut microbiota at the species level. (**d**) Gut microbiota composition at the species level. A total of 214 bacterial species were detected and the phylogenetic tree of the 16S rRNA gene sequence of each species showed the average relative abundance of these species in different populations. Relative abundance of (**e**) *Coriobacteriaceae* UCG-002, (**f**) *Lactobacillus*, (**g**) *Oscillospiraceae*, (**h**) *Lachnospiraceae*, (**i**) *Desulfovibrio fairfieldensis*, (**j**) *Clostridium* sp., (**k**) *Dorea* sp., (**l**) *Christensenella minuta*, (**m**) *Turicibacter*, (**n**) *Akkermansia*, (**o**) *UBA1819*, (**p**) *Bacteroides acidifaciens*, (**q**) *Bifidobacterium pseudolongum*, and (**r**) *Muribaculaceae*. All data are expressed by mean ± SEM (*n* = 5–6). One-way ANOVA and Tukey multiple comparison analysis were used to analyze the significance between multiple groups, * *p* < 0.05; ** *p* < 0.01; *** *p* < 0.001; **** *p* < 0.0001.

The effects of SjC supplementation on the gut microbiota composition in HFD-fed mice were explored by 16S rRNA gene sequencing of cecal contents. SjC administration slightly decreased the α-diversity index (Chao 1) of gut microbiota compared with the model group, but there was no significant difference (Figure 2a). SjC administration mainly increased the relative abundance of Desulfobacterota and decreased the relative abundance of Proteobacteria at the phylum level (Figure 2b). The PCA plots of species-level taxa show that the gut microbiota composition of mice in the HFD group is significantly different from the remaining two groups (Figure 2c) and SjC supplementation shifted the gut microbiota structure of HFD mice to that of normal-diet-fed mice, especially at the PC1 axis. A total of 214 species of gut bacteria were detected in this study and the phylogenetic tree drawn based on the 16S rRNA gene sequence showed that the relative abundances of 38 bacterial species exhibited significant differences among the three groups (*p* < 0.05) (Figure 2d). Notably, 14 of the 214 bacterial species showed high abundance and had good correlation with polysaccharide supplementation (Figure 2e–r). HFD feeding increased the relative abundances of *Desulfovibrio fairfieldensis*, *Clostridium* sp., *Dorea* sp., *Christensenella minuta*, *Turicibacter*, and *Akkermansia,* compared with the normal group, but SjC supplementation significantly suppressed the HFD-induced enrichments of these six species (Figure 2i–m). In addition, the intervention of SjC significantly suppressed the relative abundance of *Coriobacteriaceae* UCG-002 (Figure 2e) and enriched *Muribaculaceae* (Figure 2r) compared with the normal group.

### 3.4. SjC Supplementation Alleviated HFD-Induced Gut Microbiota Dysbiosis at MAG Level

**Figure 3:**
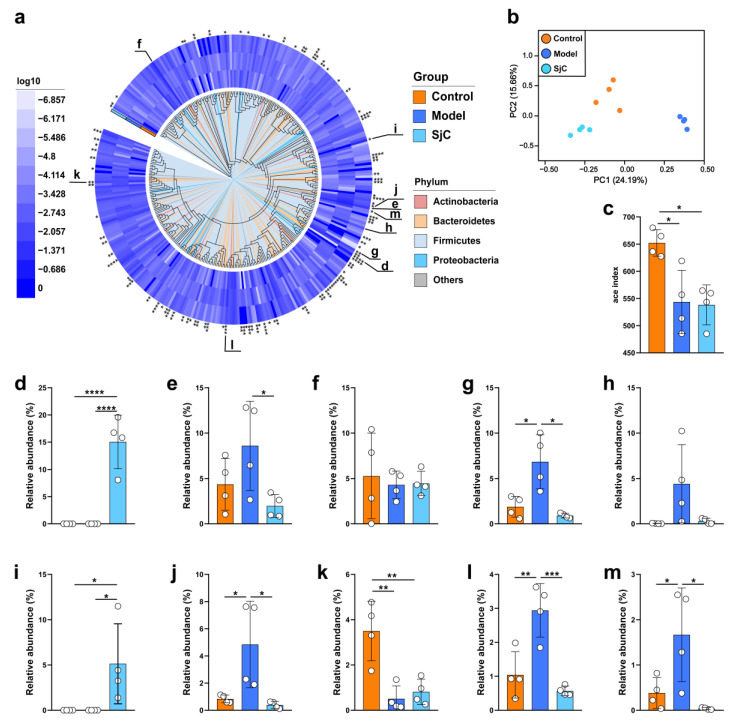
Metagenomic sequencing analysis revealed the effect of SjC administration on intestinal microbiota composition of HFD-fed mice. (**a**) Gut microbiota composition at the MAG level. The phylogenomic tree was drawn according to the core genes of each MAG. A total of 335 intestinal bacterial MAGs were detected and the average relative abundance of MAG in different populations was shown in the phylogenomic tree. (**b**) Principal component analysis (PCA) of gut microbiota composition at the species level. (**c**) Ace index of gut microbiota. Relative abundance of (**d**) *Bacillales bin.293*, (**e**) *Atopobiaceae bin.72*, (**f**) *Lachnospiraceae bin.326*, (**g**) *Desulfovibrionaceae bin.33*, (**h**) *Akkermansia muciniphila bin.80*, (**i**) *Helicobacteraceae bin.50*, (**j**) *Bifidobacterium pseudolongum bin.53*, (**k**) *Eubacteriales bin.324*, (**l**) *Bacteroidales bin.271*, and (**m**) *Atopobiaceae bacterium P1 bin.46*. All data are expressed by mean ± SEM (*n* = 4). One-way ANOVA and Tukey multiple comparison analysis were used to analyze the significance between multiple groups, * *p* < 0.05; ** *p* < 0.01; *** *p* < 0.001; **** *p* < 0.0001.

Metagenomic sequencing was also employed to investigate the effect of SjC supplementation on the gut microbiota composition in HFD-fed mice at the strain (or MAG) level. As shown in Table S1, a total of 201 Gbps of short-read data were obtained from the twelve randomly selected cecal samples and the numbers of clean reads in these samples ranged from 34,702,496 to 63,758,203. A total of 335 MAGs of gut bacteria were detected in this study and the average relative abundance of these MAGs in each group is shown in a phylogenomic tree drawn according to the core genes from each MAG. This finding indicated that the relative abundances of 126 gut bacterial MAGs showed significant differences among the three groups (*p* < 0.05) (Figure 3a). The PCA diagram of the MAGs shows that the gut microbiota composition of HFD mice is significantly different from that of the other two groups (Figure 3b). Treatment with SjC tends to restore the gut microbiota composition of HFD mice to that of normal mice at the PC1 axis. However, compared with the model group, the administration of SjC did not reverse the α-diversity index (ace) decreased (Figure 3c). Among the MAGs with significant differences among the three groups, 10 MAGs showed high relative abundance and had good correlation with SJC supplementation (Figure 3d–m). Compared with the normal group, SJC supplementation significantly increased *Bacillales bin.293*, *Helicobacteraceae bin.50*, and the relative abundance of *Eubacteria bin.324* was reduced. Compared with the model group, SJC supplementation significantly inhibited *Atopobiaceae bin.72*, *Desulfovibrionaceae bin.33*, *Bifidobacterium pseudolongum bin.53*, *Bacteroidales bin.271*, and *Atopobiaceae bacterium P1 bin.46*. In addition, compared with the model group, SjC supplementation significantly decreased the relative abundance of *Akkermania muciniphila bin.80* in some mice (Figure 3h).

### 3.5. SjC Supplementation Suppressed Amino Acid Metabolism of Gut Microbiota

**Figure 4:**
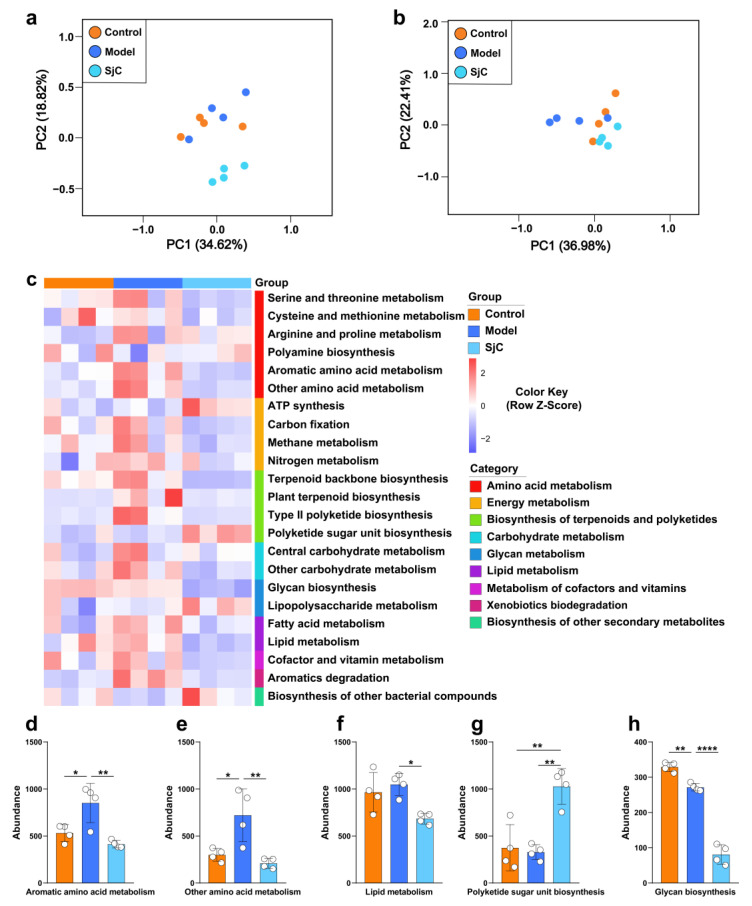
Metagenomic sequencing analysis revealed the effects of SjC supplementation on the abundance of functional genes and metabolic pathways of gut microbiota. The PCA analysis of the (**a**) functional genes and (**b**) metabolic pathways. (**c**) Metabolic pathways with significant differences (*p* < 0.05) among the groups. KEGG pathways were colored according to their functional categories. Relative abundance of (**d**) aromatic amino acid metabolism, (**e**) other amino acid metabolism, (**f**) lipid metabolism, (**g**) polyketide sugar unit biosynthesis, and (**h**) glycan biosynthesis. All data are expressed by mean ± SEM (*n* = 4). One-way ANOVA and Tukey multiple comparison analysis were used to analyze the significance between multiple groups, * *p* < 0.05; ** *p* < 0.01; **** *p* < 0.0001.

The effects of the polysaccharide, SjC, on the abundance of functional genes and metabolic pathways of the gut microbiota were explored by shotgun metagenomic sequencing. As shown in Figure 4a, the unsupervised PCA plot demonstrated that the composition of the functional genes in the gut microbiota of the control group was highly overlapped with that of the model group while the SjC group scattered far from the other groups, suggesting that SjC supplementation significantly altered the composition of the functional genes of the gut microbiota. The PCA plot of the metabolic pathway showed that the SjC group scattered closer to the control group and the model group was essentially separated in the PC2 axis from the other two groups (Figure 4b), indicating that although SjC supplementation changed the abundance of some functional genes it still maintained the metabolic homeostasis of the gut microbiota. Metabolic pathway enrichment analysis revealed the significantly altered pathways among these three groups (Figure 4c). Most amino acid metabolic pathways were enriched in HFD-fed mice and SjC supplementation suppressed the metabolic levels of these amino acids in the gut microbiota (Figure 4d,e). This trend can also be seen in some metabolic pathways in energy metabolism and lipid metabolism (Figure 4f). SjC supplementation also increased the abundance of metabolic pathways, including ATP synthesis, polyketide sugar unit biosynthesis (Figure 4g), and lipopolysaccharide metabolism. SJC supplementation also reduces glycine biosynthesis (Figure 4h).

### 3.6. SjC Supplementation Enriched Fucoidan-Degrading Gut Bacteria That Correlated with Bodyweight

Shotgun metagenomic sequencing recovered 335 MAGs with completeness ≥ 50% and contamination ≤ 10% (Table S2). According to our previous study [18], the Jensen–Shannon divergence was applied to evaluate the fucoidan degradation potential of the MAGs and the threshold was temperately defined as 0.0558. Among the 335 MAGs tested, 22 MAGs exhibited a high potential for fucoidan degradation (divergence < 0.0558), which mainly included *Firmicutes* and *Bacteroidetes* species (Table S2). Linking of 16S rRNA gene sequences with MAGs allows in-depth investigation of the role of gut bacteria during disease development and drug treatment. Our attempt successfully connected six MAGs with their 16S rRNA gene sequences (Table 1). These MAGs showed significant correlations with bodyweight. For example, bin.33, showing a high degree of similarity (divergence = 0.0454) to the gene category distribution of fucoidan-degrading bacteria, was considered to come from *Desulfovibrio*. The MAG, bin.80, showing a high degree of similarity (divergence = 0.0304) to the gene category distribution of fucoidan-degrading bacteria, was considered to come from *Akkermansia*. The draft genome of *Akkermansia* suggested that it possessed 2475 protein-coding genes and 7.1% of them were annotated as carbohydrate-active enzymes (Figure S4). In addition, the other seven gut bacterial species enriched by SjC were also found to encode many (>7.1%) polysaccharide utilization loci (Table S3). Moreover, the relative abundance of all the six tentatively identified bacterial strains are significantly correlated with mice bodyweight, including bin.33 (*Desulfovibrio*), bin.80 (*Akkermansia*), bin.271 (*Muribaculum*), and bins.120 (*Muribaculaceae uncultured*), and they may jointly mediate the occurrence of obesity. 

## 4. Discussion

In recent years, the anti-obesity and prebiotic effects of seaweed polysaccharides have gradually attracted more and more attention. Studies have shown that the gut microbiota play important roles in the beneficial effects of many dietary or medicinal polysaccharides and the interactions between the polysaccharides and gut microbiota have been considered to be the key factor affecting the pharmacological effect of polysaccharides, but the detailed genomic and functional information of the gut bacterial species that interacted with the polysaccharides are largely unknown. In this study, we prepared a macromolecular polysaccharide, SjC, from *S. japonica* and the in vivo experiment demonstrated that oral treatment with SjC could efficiently prevent HFD-induced obesity, insulin, and glucose metabolism disorders. 16S rRNA and shotgun metagenomic sequencing analysis demonstrated that SjC supplementation could prevent gut microbiota dysbiosis and suppress amino acid metabolism of the gut microbiota. A further bioinformatic study indicated that SjC enriched many gut bacterial species with fucoidan-degrading potential.

It is reported that the addition of *Desulfovibrio* to the isolated sterile mice resulted in an increase in the percentage of body fat in the small intestine and the consumption of long-chain fatty acids in the cecum contents was also significantly related to the presence of *Desulfovibrio* [32]. Nuciferine (a main bioactive component in the lotus leaf) supplementation in HFD rats can prevent weight gain, reduce lipid accumulation, and improve lipid metabolism disorder, which may be related to the reduction of the relative abundance of *Desulfovibrio* [33]. These findings suggest that *Desulfovibrio* can be studied as an important target for obesity treatment. The anti-obesity effect of SJC may be associated with the decrease of *Desulfovibrio*. *Akkermansia muciniphila* has been reported as a novel and very promising probiotic and its anti-obesity effects have been investigated in many animal models and human trials [34,35]. However, another study found that compared with the low fat diet, the HFD (Research Diets formula D12492, New Brunswick, NJ, USA) resulted in an increase of Akkermansia, which is consistent with our results [36], suggesting that the nutrient composition of the HFD has great influence on the abundance of *Akkermansia muciniphila*.

Previous studies have shown that oligosaccharides from brown seaweed *S. confusion* (SCO) can also enrich *Lactobacillus* and *Clostridium* XIVa, and inhibit the growth of *Heterobacillus*, *Bacteroides,* and *Clostridium* IV. In addition, SCO has a certain role in regulating obesity by regulating the Jnk-Irs1/Pi3k signaling pathways [37]. This suggests that SjC may also be capable of directly affecting cellular signal or metabolism to regulate obesity.

According to previous studies, gut microbiota-mediated amino acid metabolism has played an important role in gut ecology and host health [38]. Gut microbiota can inhibit the growth of the xenograft MC38 tumor by interfering with host amino acid metabolism [39]. Changes in gut microbiota structure and amino acid metabolism can also induce hyperuricemia and inflammation [40]. The study by Liu et al. showed that amino acid metabolism levels in cecal contents increased under a high fat diet, which was consistent with our findings [41]. It has also been shown that the negative effects of a high fat diet can be ameliorated by modulating the metabolism of amino acids, such as glycine, serine, and threonine [42]. However, the detailed mechanism of SjC in regulating gut microbiota-mediated amino acid metabolism needs further study.

The effects of seaweed polysaccharides on the gut microbiota in mice have been extensively investigated by 16S rRNA gene sequencing analysis and more recently, many studies employed shotgun metagenomic sequencing analysis to understand the genomic and functional diversity of the gut microbiota in mice treated with polysaccharides. However, few studies have used both 16S rRNA and shotgun metagenomic sequencing analysis to assess the effects of polysaccharides on gut bacteria, and downstream analyses are often performed separately. In this study, 16S rRNA gene sequences were preliminary linked with six MAGs, which enabled the functional analysis of specific gut bacterial species that play an important role in the anti-obesity effects. Detailed genomic characteristics can be visualized and compared with reported polysaccharide-degrading enzymes. However, the in vitro activity of *Desulfovibrio*, *Akkermansia,* and *Muribaculum* in degrading SjC needs experimental validation.

## 5. Conclusions

In conclusion, we demonstrated that the *S. japonica* polysaccharide fraction, SjC, can effectively prevent HFD-induced obesity and metabolic disorders, and alleviate gut microbiota dysbiosis. The prebiotic effects of SjC mainly involve regulating the relative abundance of *Desulfovibrio* and *Akkermansia*, and suppressing the amino acid metabolism of gut microbiota. Our findings suggest that the anti-obesity effect of SjC may be associated with the alterations of gut bacteria with fucoidan-degrading potential.

## Figures and Tables

**Table 1:** Information (Spearman’s correlation index, fucoidan-degrading potential, and blast results) about key gut bacterial species.

MAG No.	Spearman’s Correlation (MAG vs. Bodyweight). ^a^	Minimum JS Divergence ^b^	16S rRNA Blasted against MAG ^c^			
			16S rRNA No.	16S rRNA Taxonomic Classification	Identity (%)	Alignment Length
bin.293	−0.191 (*p* > 0.05)	0.0505	repseq1	*g_Alistipes; s_Alistipes_inops*	91.667	48
bin.72	0.402 (*p* > 0.05)	0.0419	repseq2	*f_Atopobiaceae*	98.795	83
bin.326	−0.136 (*p* > 0.05)	0.0493	repseq3	*f_Lachnospiraceae*	97.872	423
bin.33	0.599 (*)	0.0454	repseq4	*g_Desulfovibrio*	99.342	304
bin.53	0.492 (*)	0.0570	repseq5	*g_Desulfovibrio*	100	66
bin.50	−0.247 (*p* > 0.05)	0.0633	repseq6	*g_Helicobacter*	99.213	127
bin.324	−0.413 (*p* > 0.05)	0.0462	repseq7	*f_Oscillospiraceae; g_uncultured*	96.948	426
bin.80	0.870 (***)	0.0304	repseq8	*g_Akkermansia*	100	430
bin.271	0.682 (**)	0.0385	repseq9	*g_Muribaculum*	100	443
bin.216	−0.506 (*)	0.0329	repseq10	*g_Muribaculum*	100	443
bin.111	0.263 (*p* > 0.05)	0.0436	repseq11	*g_Turicibacter*	93.438	320
bin.256	−0.489 (*)	0.0326	repseq12	*g_Prevotellaceae_UCG-001; s_uncultured*	90.787	445
bin.322	−0.432 (*p* > 0.05)	0.0473	repseq13	*g_Alistipes*	97.065	443
bin.120	0.845 (***)	0.0340	repseq14	*g_Muribaculaceae; s_uncultured*	99.097	443
bin.46	0.514 (*)	0.0453	repseq15	*g_Blautia; s_uncultured*	88.961	154
bin.268	−0.390 (*p* > 0.05)	0.0437	repseq16	*f_Lachnospiraceae*	97.872	423
bin.161	0.567 (*)	0.0529	repseq17	*g_Lachnospiraceae_NK4A136_group*	99.291	423
bin.117	−0.475 (*p* > 0.05)	0.0581	NA			
bin.302	0.712 (**)	0.0381	repseq19	*g_Allobaculum; s_uncultured*	90.667	450
bin.219	0.594	0.0519	repseq20	*g_Clostridium_sp; s_uncultured*	98.126	427

^a^: * *p* < 0.05; ** *p* < 0.01; *** *p* < 0.001; ^b^: MAGs with a minimum JS divergence lower than 0.0558 are defined as having the fucoidan-degrading potential; ^c^: When the length of the alignment is greater than 300 bp and the identity is greater than 99%, the 16S rRNA gene sequence and the MAG are considered to come from the same strain.

## Data Availability

Data are contained within the article and Supplemental Materials.

## Supplementary Materials

The following supporting information can be downloaded at: https://www.mdpi.com/article/10.3390/foods12030665/s1, Figure S1: (a) Molecular weight determination. (b) HPLC analysis for SjC; Figure S2: The IR spectrometer of SjC; Figure S3: ^1^H-NMR spectra of SjC; Figure S4: Draft genome of *Akkermansia*; Table S1: Metagenomic sequence statistics; Table S2: Stats and JS divergence of metagenomic-assembled genomes; Table S3: The proportion of carbohydrate-active enzymes in metagenomic-assembled genomes.
