# Supplementary material for: Metagenomic Insights into the Anti-Obesity Effect of a Polysaccharide from Saccharina japonica
Source: Foods. 2023 Feb 3;12(3):665. doi: 10.3390/foods12030665 (PMC9914707; doi:10.3390/foods12030665)
Supplement: Supplementary file 1 [file foods-12-00665-s001.zip › FIgure.supplement.pdf]

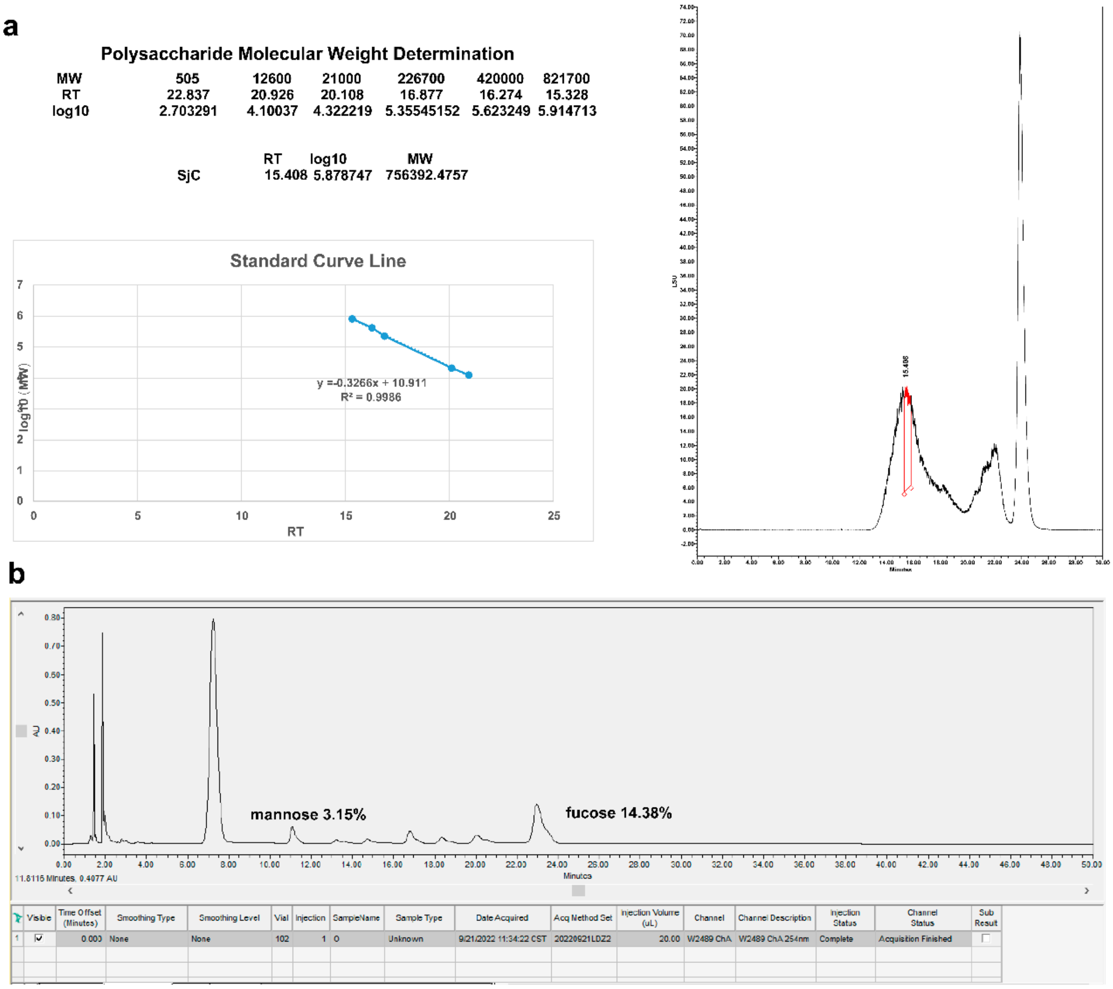

Figure S1. (a) Molecular Weight Dtermination. **(b)** HPLC analysis for SjC.

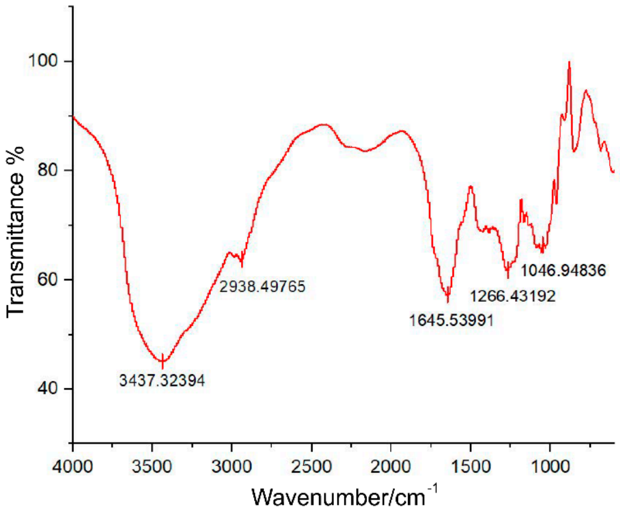

Figure S2. The IR spectrometer of SjC.

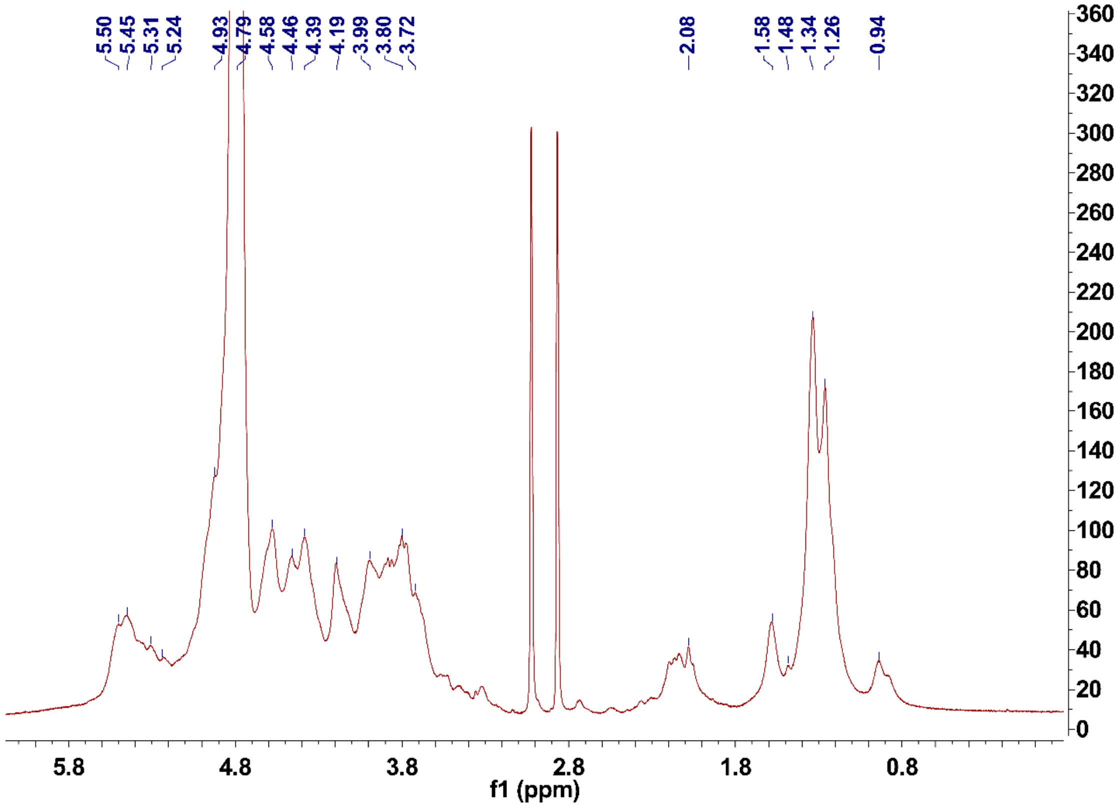

Figure S3. ^{1}H-NMR spectra of SjC.

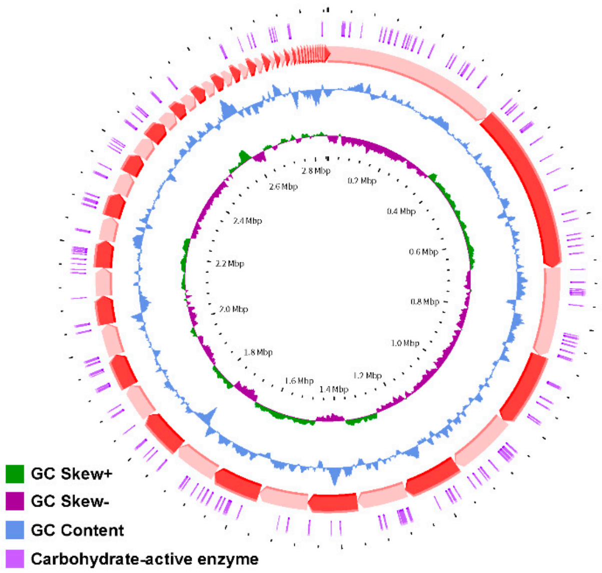

Figure S4. Draft genome of *Akkermansia*.
